# The Novel Mechanisms Concerning the Inhibitions of Palmitate-Induced Proinflammatory Factor Releases and Endogenous Cellular Stress with Astaxanthin on MIN6 β-Cells

**DOI:** 10.3390/md15060185

**Published:** 2017-06-20

**Authors:** Atsuko Kitahara, Kazuto Takahashi, Naru Morita, Toshitaka Murashima, Hirohisa Onuma, Yoshikazu Sumitani, Toshiaki Tanaka, Takuma Kondo, Toshio Hosaka, Hitoshi Ishida

**Affiliations:** Third Department of Internal Medicine, Kyorin University School of Medicine, 6-20-2 Shinkawa, Mitaka, Tokyo 181-8611, Japan; kitahara@ks.kyorin-u.ac.jp (A.K.); n-morita@ks.kyorin-u.ac.jp (N.M.); mtosh@ka3.so-net.ne.jp (T.M.); onuma@ks.kyorin-u.ac.jp (H.O.); sumitani@ks.kyorin-u.ac.jp (Y.S.); toshiaki@ks.kyorin-u.ac.jp (T.T.); takkon808@gmail.com (T.K.); toshio.hosaka@outlook.com (T.H.); ishida@ks.kyorin-u.ac.jp (H.I.)

**Keywords:** pancreatic β-cell, astaxanthin, palmitate

## Abstract

Astaxanthin, an antioxidant agent, can protect pancreatic β-cells of db/db mice from glucotoxicity and resolve chronic inflammation in adipose tissue. Nonetheless, the effects of astaxanthin on free-fatty-acid-induced inflammation and cellular stress in β-cells remain to be demonstrated. Meanwhile, palmitate enhances the secretion of pro-inflammatory adipokines monocyte chemoattractant protein-1 (MCP-1) and vascular endothelial growth factor (VEGF_120_). We therefore investigated the influence of astaxanthin on palmitate-stimulated MCP-1 and VEGF_120_ secretion in mouse insulinoma (MIN6) pancreatic β-cells. Furthermore, whether astaxanthin prevents cellular stress in MIN6 cells was also assessed. Pre-treatment with astaxanthin or with *N*-acetyl-cysteine (NAC) which is an antioxidant drug, significantly attenuated the palmitate-induced MCP-1 release through downregulation of phosphorylated c-Jun NH_2_-terminal protein kinase (JNK) pathways, and suppressed VEGF_120_ through the PI3K/Akt pathways relative to the cells stimulated with palmitate alone. In addition, palmitate significantly upregulated homologous protein (CHOP) and anti-glucose-regulated protein (GRP78), which are endoplasmic reticulum (ER) stress markers, in MIN6 cells. On the other hand, astaxanthin attenuated the increased CHOP content, but further up-regulated palmitate-stimulated GRP78 protein expression. By contrast, NAC had no effects on either CHOP or GRP78 enhancement induced by palmitate in MIN6 cells. In conclusion, astaxanthin diminishes the palmitate-stimulated increase in MCP-1 secretion via the downregulation of JNK pathways in MIN6 cells, and affects VEGF_120_ secretion through PI3K/Akt pathways. Moreover, astaxanthin can prevent not only oxidative stress caused endogenously by palmitate but also ER stress, which NAC fails to attenuate, via upregulation of GRP78, an ER chaperon.

## 1. Introduction

Astaxanthin has potent antioxidant activity and is characterized by the basic structure of a carotenoid with keto and hydroxyl groups [1,2]. Because of this structure, extension of the conjugated system causes activation of astaxanthin as a radical sponge, and astaxanthin can exert both lipophilic and hydrophilic antioxidant activities. According to the studies examining the effects of the antioxidant activity of astaxanthin, it has a wide range of properties, including improvement of physical exercise capacity as evidenced by an anti-muscle fatigue effect and an endurance-enhancing effect [3,4], prophylactic and curative effects on arteriosclerosis [5,6], influences via activation of energy metabolism including that of lipids [7], and efficacy in the treatment of eye and skin disorders such as asthenopia [8,9] and inflammatory skin diseases [10].

In addition, because astaxanthin reportedly protects pancreatic β-cells of db/db mice from glucotoxicity [11] and resolves chronic inflammation and insulin resistance in adipose tissue [12], there are high expectations about its potential clinical effects. Furthermore, astaxanthin has been confirmed to inhibit inflammation in the liver of high-fat-diet-fed mice [13], and the important finding that astaxanthin can ameliorate insulin resistance induced by TNF-α and palmitate in L6 myoblasts has also been demonstrated [14]. Nevertheless, the effects of astaxanthin on free fatty acid-induced inflammation and cellular stress in pancreatic β-cells remain as yet unknown.

Furthermore, monocyte chemoattractant protein-1 (MCP-1) and vascular endothelial growth factor (VEGF) have been confirmed to play central roles in the chronic inflammation in obese adipose tissue, which are the main contributors to insulin resistance [15,16,17,18,19]. It has also been demonstrated that secretion of MCP-1 and VEGF increases in palmitate-yielded hypertrophic 3T3-L1 adipocytes thus mimicking the hyperlipidemia frequently complicating type 2 diabetes mellitus [20]. MCP-1 secreted by hypertrophic adipocytes appears to induce recruitment of macrophages to obese adipose tissue, in which chronic inflammation may consequently be triggered [21,22]. In addition, the increased VEGF secretion by hypertrophic adipocytes has been found to augment the recruitment of macrophages into adipose tissue through enhanced angiogenesis [23]. Moreover, experimentation with db/db mice, an obese model of diabetes mellitus, has verified that administration of an antibody neutralizing VEGF inhibits not only angiogenesis but also adipogenesis, and macrophage recruitment into obese adipose tissue [23]. However, the dynamics of MCP-1 and VEGF secretion from pancreatic β-cells and the induction of chronic inflammation in islets of Langerhans in a hyperlipidemic state are essentially unknown at present.

Thus, in this study, using mouse insulinoma (MIN6) cells, we elucidated the mechanism downregulated by astaxanthin on the palmitate-induced enhancement of either MCP-1 mRNA expression and protein secretion or alternatively production of spliced VEGF_120_ lacking a heparin-binding domain [20], which are key factors of induction of chronic inflammation. Furthermore, we assessed the effects of astaxanthin on cellular stress, e.g., endogenous oxidative stress and endoplasmic reticulum (ER) stress, caused by palmitate in MIN6 cells.

## 2. Results

### 2.1. Palmitate Augments MCP-1 and VEGF_120_ Secretion by MIN6 Cells

**Figure 1:**
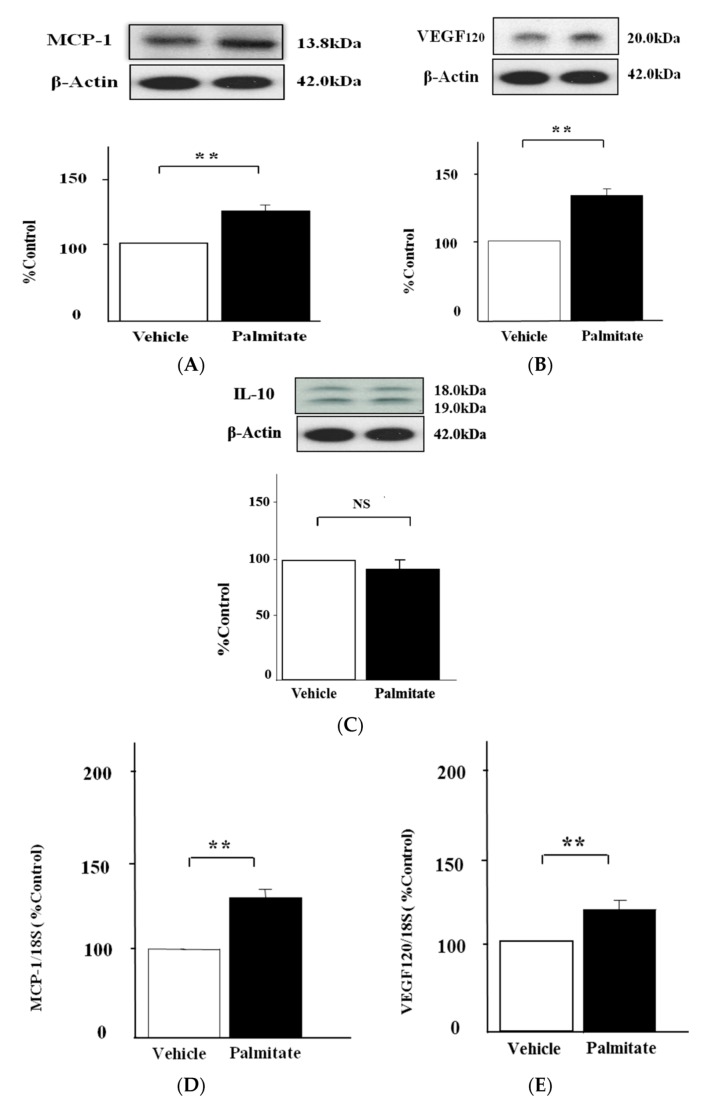
Cytokine release and expression levels on palmitate-stimulated MIN6 β-cells. MIN6 cells were stimulated with 0.3 mmol/L palmitate or ethanol vehicle alone for either 6h (**D** and **E**) or 24 h (**A**, **B** and **C**). MCP-1 (**A**), VEGF_120_ (**B**) and IL-10 (**C**) secretion by MIN6 cells was quantified by immunoblot analysis. β-Actin served as an internal control. (**A**–**C**) top: representative pictures of immunoblotting that was quantified. The mRNA levels of MCP-1 (**D**) and VEGF-A (**E**) including VEGF_120_ were measured by quantitative real-time RT-PCR. The mRNA signal for each gene was normalized to the 18S rRNA signal. Results are mean ± SEM (n = 4); ** *p* < 0.01 compared to vehicle. NS; no significant difference compared to vehicle.

Palmitate markedly increased MCP-1 secretion by 1.3-fold in the analysis by western blotting (*p* < 0.01; Figure 1A). In addition, VEGF_120_ release was enhanced 1.6-fold by treatment with palmitate (*p* < 0.01; Figure 1B). As shown in Figure 1D,E, MCP-1 mRNA expression was increased 1.3-fold by palmitate (*p* < 0.01), and VEGF-A including VEGF_120_ was upregulated 1.4-fold (*p* < 0.01). Meanwhile, palmitate had no effect on IL-10 release (Figure 1C).

### 2.2. Astaxanthin Reverses Palmitate-Induced Enhancement of MCP-1 and VEGF_120_ Secretion

**Figure 2:**
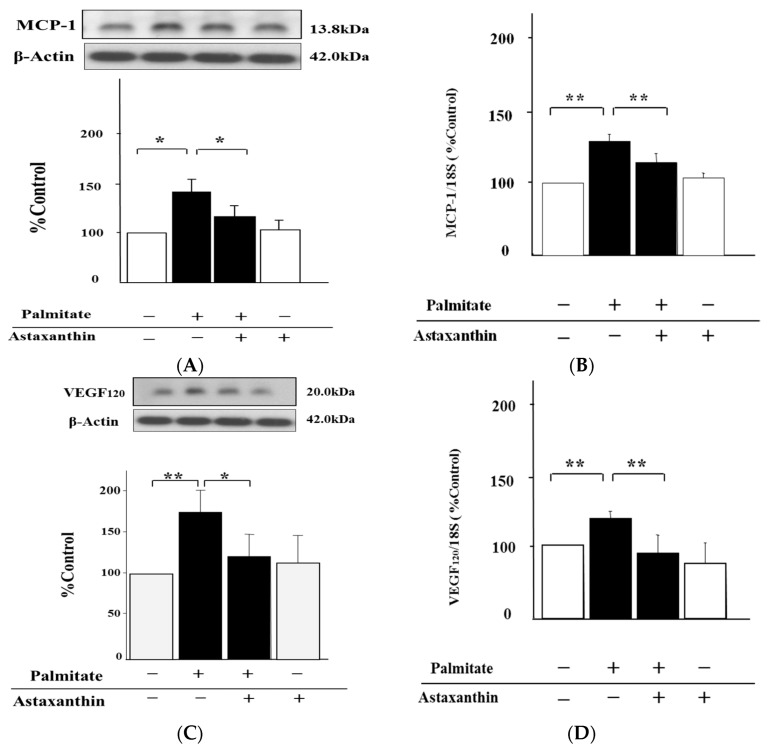
Astaxanthin reverses MCP-1 and VEGF_120_ upregulation by palmitate. MIN6 β-cells were pretreated with 10 µmol/L astaxanthin for 20 min, and then these cells were treated with 0.3 mmol/L palmitate for either 6 h (**B** and **D**) or 24 h (**A** and **C**), with or without astaxanthin. MCP-1 (**A**) and VEGF_120_ (**C**) secretion was analyzed by quantitative immunoblots. MCP-1 (**B**) and VEGF_120_ (**D**) mRNA expression levels were measured by real-time PCR. β-Actin was assessed as an internal control. (**A**) and (**C**) top: representative pictures of immunoblotting that was quantified. The mRNA signal for each gene was normalized to the 18S rRNA signal. Results are mean ± SEM (n = 4); * *p* < 0.05; ** *p* < 0.01 compared to the corresponding controls.

We tested whether the treatment with astaxanthin could affect MCP-1 and VEGF_120_ expression in MIN6 cells treated with palmitate. Pre-treatment with astaxanthin significantly diminished the release of MCP-1 by 33% and VEGF_120_ by 30% relative to the cells stimulated with palmitate alone (*p* < 0.05, respectively; Figure 2A,C). On the other hand, there were no significant changes in MCP-1 and VEGF_120_ release under the influence of astaxanthin treatment alone (Figure 2A,C). Furthermore, the significant enhancement of both MCP-1 and VEGF-A (including VEGF_120_) mRNA expression by treatment with palmitate was attenuated by astaxanthin by 18% and 34%, respectively (*p* < 0.01; Figure 2B,D). In contrast, astaxanthin alone failed to increase MCP-1 and VEGF-A mRNA expression (Figure 2B,D).

### 2.3. NAC, an Antioxidant Agent, Can Inhibit Palmitate-Stimulated MCP-1 and VEGF_120_ Secretion

**Figure 3:**
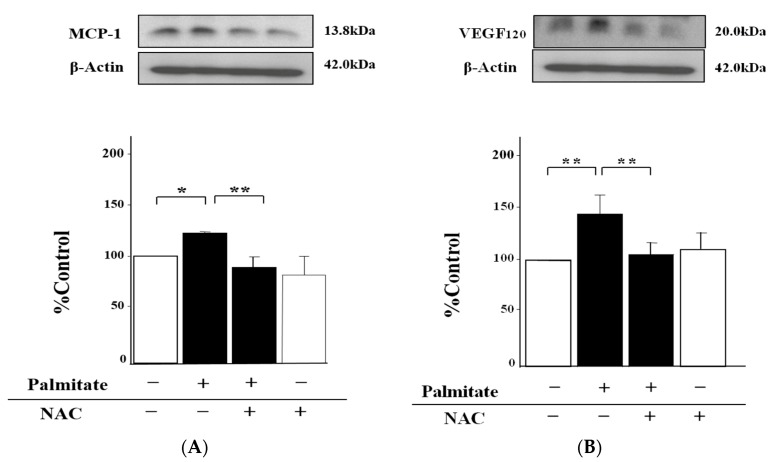
NAC reduces palmitate-induced MCP-1 and VEGF_120_ release from MIN6 cells. MIN6 cells were pretreated with 1 mmol/L NAC or vehicle (dimethyl sulfoxide) for 20 min, and then these cells were treated with 0.3 mmol/L palmitate for 24 h with or without NAC. MCP-1 (**A**) or VEGF_120_ (**B**) secretion was measured by quantitative immunoblots. β-Actin served as an internal control. (**A** and **B**) top: representative pictures of immunoblotting that was quantified. Results are mean ± SEM (n = 4); * *p* < 0.05; ** *p* < 0.01 compared to the corresponding controls.

NAC lowered the palmitate-induced increase of the MCP-1 release by 22% compared with MIN6 cells exposed to palmitate alone (*p* < 0.01; Figure 3A). Moreover, the enhancement of VEGF_120_ secretion by the stimulation with palmitate was reduced by NAC treatment by 27% (*p* < 0.01; Figure 3B).

On the other hand, treatment with NAC alone was unable to influence either MCP-1 or VEGF_120_ secretion (Figure 3A,B).

### 2.4. Astaxanthin Can Inhibit Oxidative Stress

**Figure 4:**
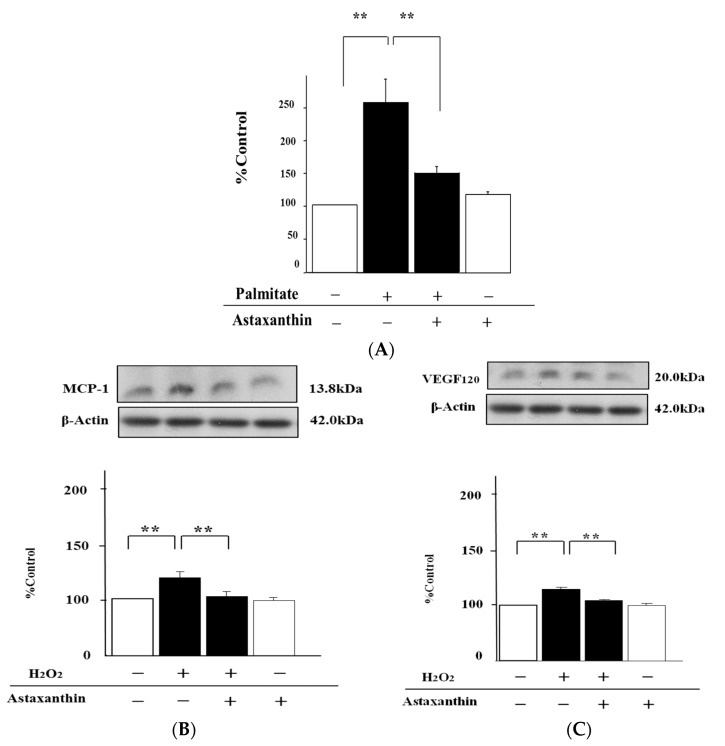
Astaxanthin attenuates palmitate-induced upregulation of hydroperoxides and the release of both MCP-1 and VEGF_120_ increased by H_2_O_2_. MIN6 β-cells pretreated with 10 µmol/L astaxanthin for 20 min were stimulated with 0.3 mmol/L palmitate (**A**) or 300 ng/mL H_2_O_2_ (**B** and **C**) for 24 h. Hydroperoxide content in MIN6 cells was measured by means of the Free Radical Elective Evaluator system (**A**). Results are mean ± SEM (n = 4). The release of MCP-1 (**B**) and VEGF_120_ (**C**) was quantified by immunoblot analysis. β-Actin was assessed as an internal control. (**B**) and (**C**) top: representative pictures of immunoblotting that was quantified. Results are mean ± SEM (n = 4); ** *p* < 0.01 compared to the corresponding controls.

The intracellular concentration of hydroperoxides, a marker of endogenous oxidative stress, in cells treated with palmitate was augmented 2.5-fold (*p* < 0.01; Figure 4A). In contrast, astaxanthin was able to attenuate this upregulation of hydroperoxides by 30% compared with palmitate-stimulated cells (*p* < 0.01; Figure 4A). Meanwhile, exogenous H_2_O_2_ increased the MCP-1 release 1.4-fold (*p* < 0.01; Figure 4B), and the pre-treatment with astaxanthin suppressed this effect by 30% relative to MIN6 cells treated with H_2_O_2_ alone (*p* < 0.01; Figure 4B).

As with MCP-1, astaxanthin also decreased VEGF_120_ secretion by 30% (*p* < 0.01; Figure 4C), whereas H_2_O_2_ alone clearly enhanced this section 1.3-fold (*p* < 0.01; Figure 4C).

### 2.5. The MCP-1 Release by MIN6 Cells Treated with Palmitate Is Increased via JNK Pathways

**Figure 5:**
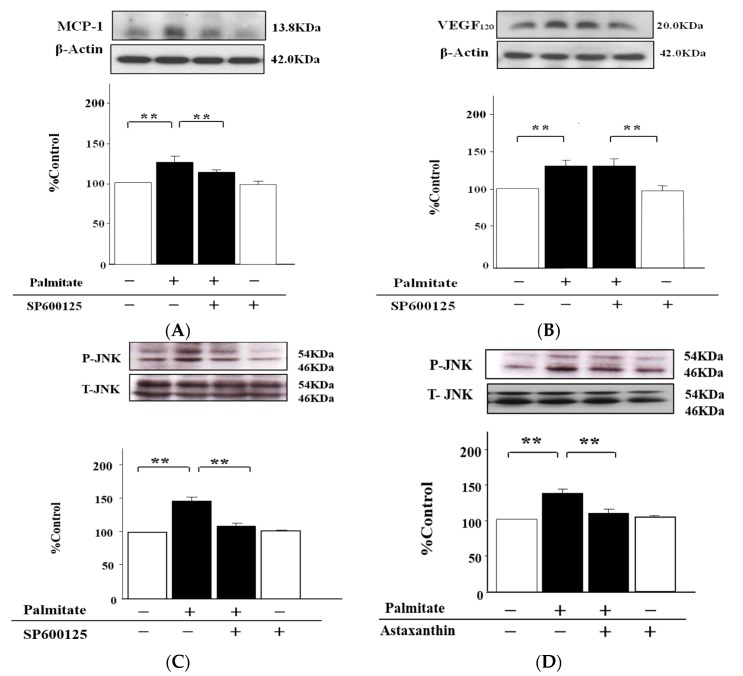
The MCP-1 release from MIN6 cells is enhanced via JNK, but the JNK pathway is unrelated to VEGF_120_ secretion. MIN6 cells were pretreated with 10 µmol/L SP600125, 10 µM astaxanthin, or vehicle (dimethyl sulfoxide) alone for 20 min. Then, the cells were incubated with 0.3 mmol/L palmitate or vehicle (ethanol) alone for 24 h with or without either SP600125 (**A**, **B** and **C**) or astaxanthin (**D**). MCP-1 secretion (**A**) and VEGF_120_ secretion (**B**) were then quantified by immunoblotting with β-actin as an internal control. JNK phosphorylation on Thr183/Tyr185 (**C** and **D**) was also quantified by immunoblot analysis. Phospho-JNK was normalized to total JNK protein. (**A**–**D**) top: representative pictures of immunoblotting that was quantified. Results are mean ± SEM (n = 4). ** *p* < 0.05; ** *p* < 0.01 compared to the corresponding controls.

Next, we determined whether MAPKs pathways are involved in the MCP-1 release. The treatment with JNK inhibitor SP600125 (10 µmol/L) significantly reduced the increased MCP-1 secretion by 38% relative to the cells stimulated with palmitate alone (*p* < 0.05; Figure 5A). On the other hand, SP600125 had no effects on palmitate-induced enhancement of VEGF_120_ secretion (Figure 5B).

Moreover, we evaluated phosphorylation levels of JNK. JNK phosphorylation on Thr183/Tyr185 was significantly increased 1.6-fold by treatment with palmitate (*p* < 0.01; Figure 5C). However, SP600125 significantly attenuated the palmitate-stimulated JNK phosphorylation by 25%, (*p* < 0.01; Figure 5C), and astaxanthin as well as the SP600125 also inhibited the enhanced JNK phosphorylation by 24% (*p* < 0.01; Figure 5D). Meanwhile, astaxanthin alone had no effects on JNK phosphorylation (Figure 5D).

### 2.6. Palmitate Upregulates VEGF_120_ Secretion via PI3K Pathways

**Figure 6:**
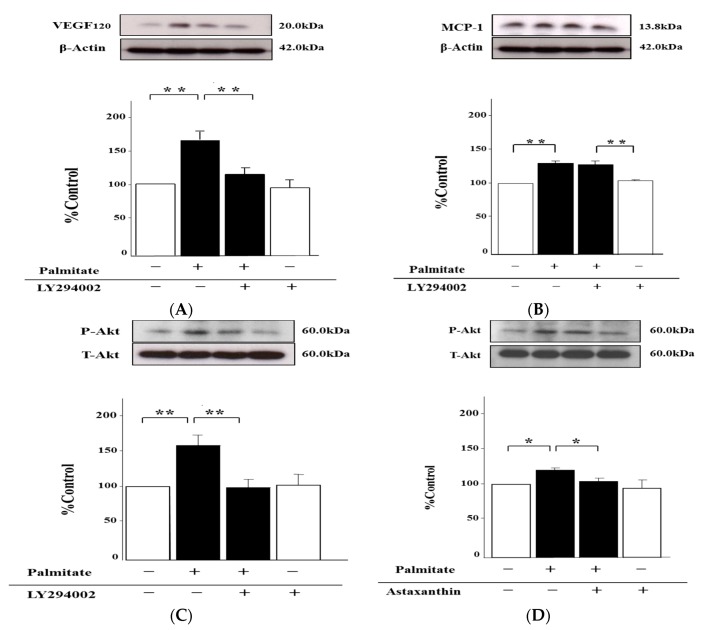
Involvement of PI3K (phosphatidylinositol 3-kinase) pathways in VEGF_120_ secretion by MIN6 β-cells. MIN6 cells were pretreated with 50 µmol/L LY294002, 10 µmol/L astaxanthin, or vehicle (dimethyl sulfoxide) alone for 20 min. Then, the cells were stimulated with 0.3 mmol/L palmitate or vehicle (ethanol) alone for 24 h with or without either LY294002 (**A**, **B** and **C**) or astaxanthin (**D**). VEGF_120_ (**A**) and MCP-1 secretion (**B**) was analyzed by immunoblotting. β-Actin was assessed as an internal control. Akt phosphorylation on Ser473 under the influence of LY294002 or astaxanthin is shown in (**C**) and (**D**) as immunoblotting. Phospho-Akt was normalized to total Akt protein. (**A**–**D**) top: representative pictures of immunoblotting that was quantified. Results are mean ± SEM (n = 4); * *p* < 0.05; ** *p* < 0.01 compared to the corresponding controls.

To examine the signaling pathways involved in the palmitate-stimulated increase of VEGF_120_ secretion, we used LY294002, an inhibitor of phosphatidylinositol 3-kinase (PI3K), which has been reported to repress VEGF_120_ release by adipocytes [23]. LY294002 markedly diminished the palmitate-induced increase of VEGF_120_ secretion from MIN6 cells by 27% (*p* < 0.01; Figure 6A). Nonetheless, there were no effects of treatment with LY294002 on palmitate-induced MCP-1 increase (Figure 6B). In addition, LY294002 was obviously able to attenuate Akt phosphorylation on Ser473, which was augmented by the treatment with palmitate, by 30% (*p* < 0.01; Figure 6C). As with LY294002, astaxanthin inhibited the enhancement of Akt phosphorylation with the exposure of palmitate by 20% (*p* < 0.01; Figure 6D). However, astaxanthin alone failed to impact the Akt phosphorylation (Figure 6D).

### 2.7. Astaxanthin Can Eliminate ER Stress via the Enhancement of GRP78 Expression

**Figure 7:**
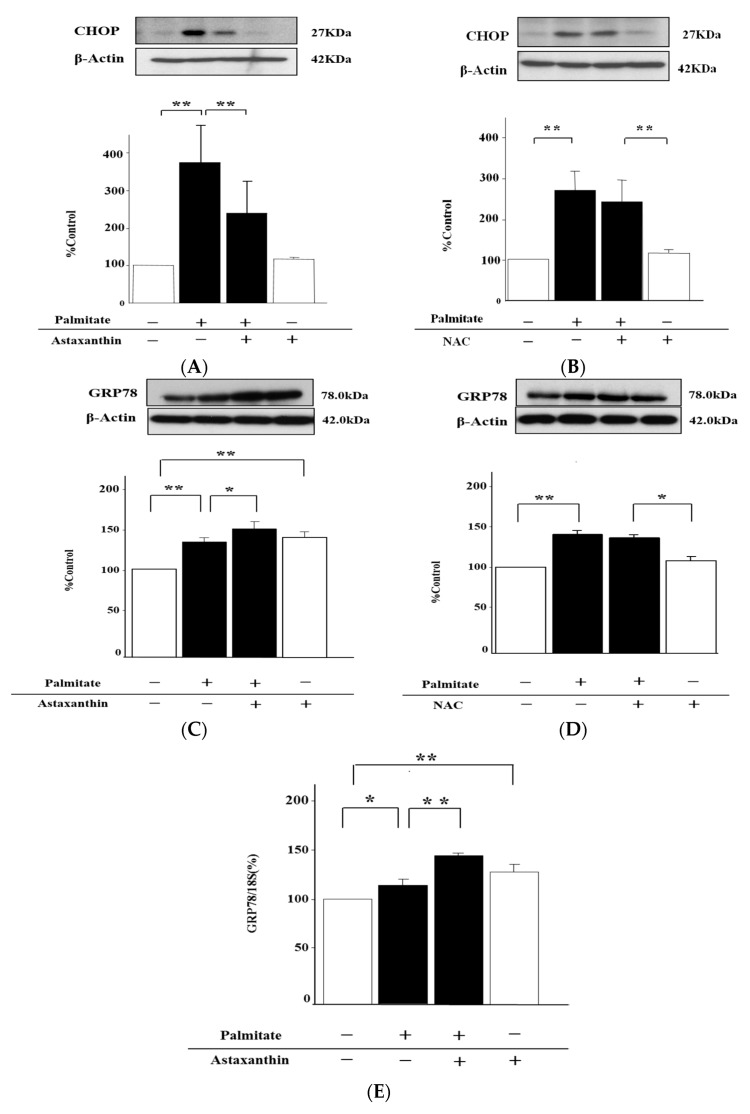
Astaxanthin diminished ER stress via the increase of GRP78, but NAC cannot, in MIN6 cells. MIN6 cells were pretreated with 10 µmol/L astaxanthin (**A**, **C** and **E**), 1 mM NAC (**B** and **D**), or vehicle (dimethyl sulfoxide) alone for 20 min. Then, the cells were incubated with 0.3 mmol/L palmitate or vehicle (ethanol) alone for 24 h. CHOP (**A** and **B**) and GRP78 contents (**B** and **D**) were measured by immunoblotting. β-Actin served as an internal control. (**A**)–(**D**) top: representative pictures of immunoblotting that was quantified. The GRP78 mRNA level (**E**) was assessed using Digital PCR System. The mRNA signal was normalized to the 18S rRNA signal. Results are mean ± SEM (n = 4); * *p* < 0.05; ** *p* < 0.01 compared to the corresponding controls.

Moreover, we tested whether astaxanthin has effects against ER stress. Palmitate significantly upregulated CHOP, 3.6 and 2.7-fold, (*p* < 0.01; Figure 7A,B), and GRP78.

1.3 and 1.2-fold, (*p* < 0.01; Figure 7C,D), which are ER stress markers, in MIN6 cells. On the other hand, astaxanthin was able to attenuate the palmitate-stimulated increase in CHOP content by 36% (*p* < 0.01; Figure 7A), but alone had no effect on CHOP (Figure 7A). NAC failed to inhibit palmitate-stimulated upregulation of CHOP and GRP78 in MIN6 cells (Figure 7B,D). Contrary to CHOP content, the reinforcement of GRP78 by palmitate was further increased 1.2-fold by astaxanthin (*p* < 0.01; Figure 7C), and then astaxanthin treatment alone also increased the GRP78 content 1.3-fold (*p* < 0.01; Figure 7C). Accordingly, we explored the influence of astaxanthin on GRP78 mRNA expression. This expression was markedly enhanced 1.2-fold by treatment with astaxanthin (*p* < 0.01; Figure 7E). Furthermore, the palmitate-stimulated upregulation of GRP78 mRNA was increased 1.2-fold by astaxanthin (*p* < 0.01; Figure 7E).

## 3. Discussion

Palmitate reportedly causes cellular dysfunction in pancreatic β-cells [24,25], and MCP-1 and VEGF have been confirmed to promote chronic inflammation by recruiting macrophages into hypertrophic adipose tissue. Furthermore, in our previous study, we have demonstrated that 3T3-L1 adipocytes stimulated with palmitate increase the secretion of MCP-1 and VEGF_120_ [20].

Thus, we first examined palmitate effects on mRNA expression and secretion of both MCP-1 and VEGF_120_ in β-cells. Stimulation with palmitate led to significant increases in MCP-1 mRNA expression and secretion in MIN6 cells, as was the case for VEGF_120_. These results indicate that palmitate controls the expression levels of MCP-1 and VEGF_120_ at the transcription level.

According to all these results, palmitate increases the secretion of VEGF_120_, so that angiogenesis in the islets of the pancreas can be enhanced. Additionally, on the basis of this angiogenesis, the increase in palmitate-stimulated MCP-1 release by β-cells is able to induce macrophage infiltration into the islets; this process might consequently trigger inflammation in these islets and ultimately cause pancreatic β-cell dysfunction.

On the other hand, astaxanthin clearly reduced both MCP-1 and VEGF_120_ mRNA expression and release which had been significantly increased by palmitate. According to these results, we can hypothesize that palmitate enhances both the secretion of MCP-1 and VEGF_120_ by β-cells and induces macrophage infiltration into the islets of pancreas, which might consequently trigger inflammation in these islets and ultimately cause pancreatic β-cell dysfunction. In contrast, astaxanthin appears to protect β-cells from this damage, the dysfunction of β-cells, by suppressing the palmitate-induced release of MCP-1 and VEGF_120_.

Next, because we had previously demonstrated that palmitate augments MCP-1 and VEGF_120_ release by increasing oxidative stress in adipocytes [20], the effects of palmitate and astaxanthin on cellular stress in β-cells were examined. Although palmitate increased oxidative stress, which is a major form of cellular stress, in MIN6 cells, the antioxidant astaxanthin, as expected, attenuated the oxidative stress that had been increased by palmitate. In addition, hydrogen peroxide, which is one of the reactive oxygen species (ROS), enhanced both MCP-1 and VEGF_120_ secretion by MIN6 cells. On the other hand, astaxanthin as well as NAC, another major antioxidant agent, can apparently inhibit the palmitate-induced increase in the MCP-1 and VEGF_120_ release by attenuating oxidative stress. Accordingly, we can theorize that astaxanthin can reduce the MCP-1 and VEGF_120_ release by diminishing oxidative stress, whereas palmitate enhances, though only partially, the secretion of MCP-1 and VEGF_120_ through increasing oxidative stress.

In addition, we verified the involvement of the JNK and PI3K/Akt pathways, which are intracellular signal transduction pathways acting downstream of oxidative stress [26,27]. The increase of MCP-1 secretion in response to stimulation with palmitate was significantly reduced by the treatment with SP600125, a JNK-specific inhibitor, but there were no effects of the treatment with LY294002, an inhibitor of PI3K/Akt, on the palmitate-induced MCP-1 release. Additionally, the phosphorylation of JNK augmented by palmitate was also clearly attenuated by both astaxanthin and SP600125. Given these results, we supposed that palmitate activates the JNK pathways probably by increasing oxidative stress, thereby enhancing theMCP-1 release. On the contrary, VEGF_120_ secretion reinforced by palmitate was repressed by LY294002, while SP600125 had no effect on the palmitate-stimulated increase in the VEGF_120_ release. Moreover, the enhanced phosphorylation of Akt, which was caused by the treatment with palmitate, was significantly diminished by astaxanthin and LY294002. Therefore, palmitate may activate PI3K/Akt pathways via augmenting oxidative stress no less than palmitate can activate the JNK pathway, which is involved in the MCP-1 release, thus resulting in the increase of VEGF_120_ secretion.

Furthermore, besides oxidative stress, analyzed the effects of astaxanthin on ER stress, the other major cellular stress. Although it has previously been suggested that astaxanthin may attenuate ER stress in the liver [28], its effects on pancreatic β-cell stress remain to be elucidated. Palmitate, a saturated fatty acid, exacerbated ER stress, as well as oxidative stress, in MIN6 cells as previously reported [29,30,31,32]. Interestingly, an antioxidant, NAC, failed to suppress the ER stress enhanced by palmitate, whereas astaxanthin, another antioxidant, attenuated this enhancement of ER stress in addition to oxidative stress. This result suggests that astaxanthin not only has an antioxidant function but also broadly exerts anti-cellular-stress actions. We further investigated the mechanisms underlying this action. Palmitate enhanced the expression levels of CHOP and GRP78, which are ER stress markers [33,34]. Astaxanthin significantly suppressed palmitate-enhanced CHOP protein expression but further upregulated palmitate-induced GRP78. These data show that astaxanthin can promote correction of the folding of abnormal proteins that have been elevated by palmitate treatment and can attenuate ER stress by enhancing the expression of GRP78, which is an ER chaperone [35]. Astaxanthin is, therefore, likely to suppress palmitate-enhanced CHOP expression but to enhance GRP78 expression. Concerning this upregulation of GRP78 stimulated with astaxanthin, it seems that our study is the first report of this phenomenon. ER stress may be apt to happen due to lipotoxicity in MIN6 cells, because β-cells are more vulnerable to cellular stress [36,37]. Nonetheless, further studies may need to be conducted focusing on this issue, including associated transcription factors.

**Figure 8:**
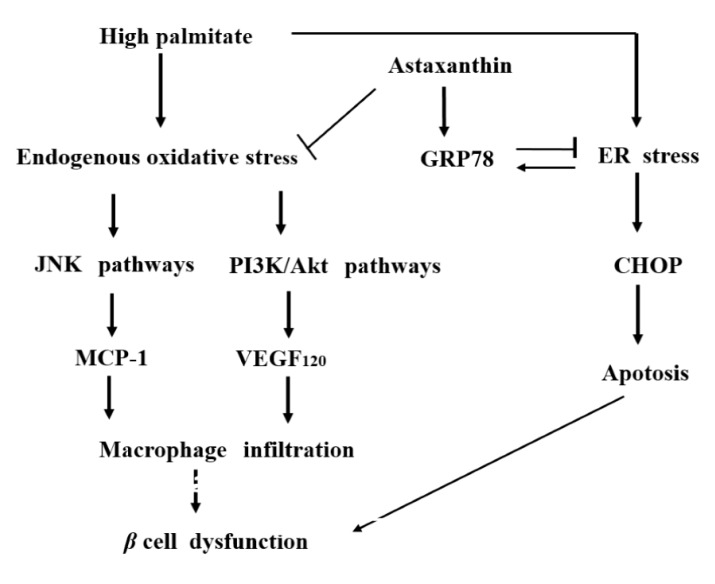
The schema of putative mechanisms behind endogenous MCP-1 and VEGF-induced low-grade chronic inflammation and insulin resistance in pancreatic β-cells in vivo, and of prevention thereof by astaxanthin. High concentration of palmitate increases endogenous oxidative stress, allowing for the MCP-1 release to be enhanced through the JNK pathways activated by oxidative stress; VEGF_120_ release from these cells nevertheless can be upregulated via activation of distinct signaling of the PI3K/Akt pathways. Moreover, the increase of palmitate-stimulated MCP-1 and VEGF_120_ release by β-cells can induce macrophage infiltration and causes pancreatic β-cell dysfunction. Astaxanthin inhibits the palmitate-increased MCP-1 and VEGF_120_ secretion through the attenuation of endogenous oxidative stress and its downstream JNK and PI3K/Akt activation, resulting in prevention of β-cell dysfunction induced by palmitate. In addition, palmitate enhances ER stress, which is manifested as enhancement of CHOP and GRP78 expression. On the other hand, astaxanthin reduces this CHOP augmentation, but further upregulates GRP78; therefore, astaxanthin can protect cells from ER stress-mediated apoptosis and subsequently also prevent β-cell dysfunction.

In conclusion, this study is the first to demonstrate that astaxanthin inhibits the secretion of MCP-1 and VEGF_120_ by pancreatic β-cells; these proteins are increased in the hyperlipidemic state frequently associated with type 2 diabetes mellitus. Consequently, it is likely that astaxanthin relieves chronic inflammation of the pancreas and prevents pancreatic β-cell damage. Moreover, this study confirmed that increased MCP-1 secretion by β-cells in the hyperlipidemic state is involved in the reinforcement of oxidative stress and of JNK pathways activated by this oxidative stress. It was also demonstrated that hyperlipidemia-stimulated VEGF_120_ secretion by pancreatic β-cells is associated with oxidative stress, and PI3K/Akt pathways are activated downstream of the oxidative stress. Accordingly, it appears that astaxanthin inhibits activation of both JNK and PI3K/Akt pathways by attenuating oxidative stress, so that increased secretion of MCP-1 and VEGF_120_ by β-cells in the hyperlipidemic state is suppressed, ultimately preventing pancreatic β-cell damage (Figure 8).

In addition, because astaxanthin may reduce ER stress, as well as oxidative stress, by enhancing GRP78 expression in β-cells, we can reasonably suppose that astaxanthin functions as more than a mere antioxidant agent and exert an anti-cellular-stress action against a wide range of cellular stresses. Thus, astaxanthin can be regarded as a potentially important factor that not only prevents pancreatic β-cell damage through antioxidative action, but also eliminates various types of cellular stress and thereby broadly protects pancreatic β-cells and islets (Figure 8). Presumably, astaxanthin may play an important role in the treatment of diabetes mellitus and hyperlipidemia by restoring insulin secretion and insulin sensitivity. Additionally, this possibility seems to merit further research aimed at utilizing this compound as a preventive pharmacotherapy in the future practice.

## 4. Materials and Methods

### 4.1. Reagents

XF Palmitate-BSA FAO Substrate was purchased from Seahorse Bioscience (North Billerica, MA, USA), and astaxanthin was obtained from Fuji Chemical Industry Co., Ltd. (Toyama, Japan). SP600125 and LY294002 were purchased from A.G. Scientific, Inc. (San Diego, CA, USA). Antibodies against CCAAT/enhancer-binding protein (C/EBP) homologous protein (CHOP), Akt1/2/3 (phospho Tyr315/316/312) and phosphorylated c-Jun NH_2_-terminal protein kinase (JNK) were obtained from Santa Cruz Biotechnology, Inc. (Santa Cruz, CA, USA)., and antibody against phosphorylated Akt1/2/3 from Assay Designs (Ann Arbor, MI, USA). Antibodies against MCP-1, VEGF_120_, IL-10, JNK, and anti-glucose-regulated protein (GRP78)/binding immunoglobulin protein (Bip) were obtained from R&D Systems (Minneapolis, MN, USA), and *N*-acetyl-cysteine (NAC), hydrogen peroxide (H_2_O_2_), and the antibody against β-Actin from Sigma-Aldrich (St. Louis, MO, USA).

### 4.2. Preparation of MIN6 Cells

Mouse MIN6 pancreatic β-cells were grown in DMEM (high glucose, without sodium pyruvate) containing 25 mmol/L glucose supplemented with 15% fetal calf serum (FCS), 50 U/mL penicillin, 50 μg/mL l-glutamine,10 μl/L 2-mercaptoethanol, and 100 mmol/L sodium pyruvate in a humidified atmosphere containing 5% of CO_2_ at 37 °C. At confluence, the cells were used for experiments.

### 4.3. Treatment of MIN6 Cells

XF Palmitate-BSA FAO Substrate (Seahorse Bioscience) was dissolved in an FCS-free medium, then the final concentration of palmitate in the medium was adjusted to 0.3 mmol/L. At 20 min before palmitate stimulation, 10 µmol/L astaxanthin, 1 mmol/L NAC, 10 μmol/L SP600125, or 10 μmol/L LY294002 was added in the culture medium. At 24h after the exposure to palmitate, the cell lysates and medium were subjected to various experiments.

In other experimental series, MIN6 cells were pretreated with astaxanthin, and then after 20 min, 0.3 mmol/L H_2_O_2_ was added into the culture medium. At 24 h after H_2_O_2_ administration, MCP-1 and VEGF_120_ secretion were analyzed by immunoblotting.

### 4.4. Immunoblotting

MIN6 cells were lysed in SDS sample buffer containing 1% Phosphatase Inhibitor Cocktail (Nacalai Tesque, Kyoto, Japan), were sonicated, and centrifuged. The resulting supernatants were boiled in the presence of 50 mmol/L dithiothreitol. To measure secreted proteins, the supernatant of the culture medium with the cells was also boiled in SDS sample buffer containing 1% Phosphatase Inhibitor Cocktail with 50 mmol/L dithiothreitol. Boiled samples were subjected to SDS PAGE, and transferred onto polyvinylidene difluoride membranes (Bio Craft, Tokyo, Japan). Membranes were incubated with primary antibodies as described in the section Reagents, and thereafter with horseradish peroxidase-conjugated secondary antibody. Protein bands were visualized with chemiluminescence reagents according to the manufacturer’s protocol (Amersham, Little Chalfont, Buckinghamshire, UK). Bands were scanned and analyzed with NIH Image software. Protein band intensities under basal conditions were set to 100% for normalization purposes.

### 4.5. Real-Time Quantitative PCR

Using the RNA queous^®^-4PCR kit (Ambion, Austin, TX, USA), total RNA was extracted from MIN6 cells according to the manufacturer’s instructions, and then was reverse-transcribed to cDNA. Real-time quantitative PCR was conducted using the 7300 real-time PCR system (Applied Biosystems, Foster City, CA, USA). The following primers and probes were ordered from Applied Biosystems: VEGF-A (Mm03015192_m1) and MCP-1 (Mm00441242_m1). The mRNA signal was normalized to the 18S rRNA signal. The mean value of triplicates was used for comparison of mRNA levels.

### 4.6. Quantstudio 3D dPCR

Total RNA was extracted by the RNAqueous^®^-4PCR kit (Ambion, Austin, TX, USA), then was used to synthesize cDNA. The cDNA was analyzed by the™ QuantStudio^®^ 3D Digital PCR System (Thermo Fisher Scientific, Waltham, MA, USA). The cDNA was first loaded onto the chips using the QuantStudio^®^ 3D Digital PCR Chip Loader with a mixture comprising 2× Quantstudio^®^ 3D digital PCR mastermix, and also 300 nmol/L of GRP78 primers and probes (Mm00517691_m1; Applied Biosystems, Foster City, CA, USA). Next, the chips were sealed and loaded onto a GeneAMPR PCR system 9700 (Applied Biosystems, Foster City, CA, USA), and then the following cycling conditions were used: 96 °C for 10 min, followed by 39 cycles of 60 °C for 2 min and 98 °C for 30 s, and a final extension at 60 °C for 2 min. Finally, the end-point fluorescence of the segmentation processes on the chips was analyzed by transferring the chips to the measurement unit (application version 1.1.3, algorithm version 0.13, Applied Biosystems, Foster City, CA, USA). In addition, the mRNA signal of GRP78 was normalized to18S rRNA.

### 4.7. Quantification of Hydroperoxides

MIN6 cells were incubated as discussed previously, supernatants were removed, and the cells were washed three times with PBS. The cells were lysed in a buffer consisting of 0.5 mmol/L Tris-HCl (pH 7.4), 1.5 mmol/L NaCl, 2.5% deoxycholic acid, and 10% Nonidet P-40. Lysates were centrifuged for 10 min at 15,000 *g* and 4 °C; the supernatants were assayed for intracellular endogenous hydroperoxides by the Free Radical Elective Evaluator system (Diacron, Grosseto, Italy) according to the manufacturer’s protocol. Hydroperoxide units of Carratelli units were adjusted to intracellular total protein contents.

### 4.8. Statistical Analysis

Statistical analysis was performed by unpaired *t*-test or by analysis of variance (ANOVA). Results are expressed as mean ± SEM and *p* < 0.05 was considered statistically significant.

## Supplementary Materials

The following are available online at www.mdpi.com/1660-3397/15/6/185/s1. Figure S1. Palmitate (0.3 mmol/L) can not induce endoplasmic reticulum stress in hypertrophied 3T3-L1 adipocytes, in contrast to in MIN6 β-cells．Mature 3T3-L1 adipocyte were preloaded with 0.3 mmol/L palmitate or ethanol vehicle alone for 24 h (A, B). A and B: Intraceller CHOP (**a**) and GRP78 (**b**) protein content were quantified by immunoblot analysis. β-Actin served as an internal control. Results are means ± SE (n = 4). ** *p* < 0.01 compared to vehicle. NS; no significant difference compared to vehicle.
