# Supplementary material for: The Novel Mechanisms Concerning the Inhibitions of Palmitate-Induced Proinflammatory Factor Releases and Endogenous Cellular Stress with Astaxanthin on MIN6 β-Cells
Source: Mar Drugs. 2017 Jun 20;15(6):185. doi: 10.3390/md15060185 (PMC5484135; doi:10.3390/md15060185)
Supplement: Supplementary file 1 [file marinedrugs-15-00185-s001.pdf]

Supplementary Materials: The Novel Mechanisms Concerning the Inhibitions of Palmitate-Induced Proinflammatory Factor Releases and Endogenous Cellular Stress with Astaxanthin on MIN6 β-cells

Atsuko Kitahara, Kazuto Takahashi , Naru Morita, Toshitaka Murashima, Hirohisa Onuma, Yoshikazu Sumitani, Toshiaki Tanaka, Takuma Kondo, Toshio Hosaka and Hitoshi Ishida

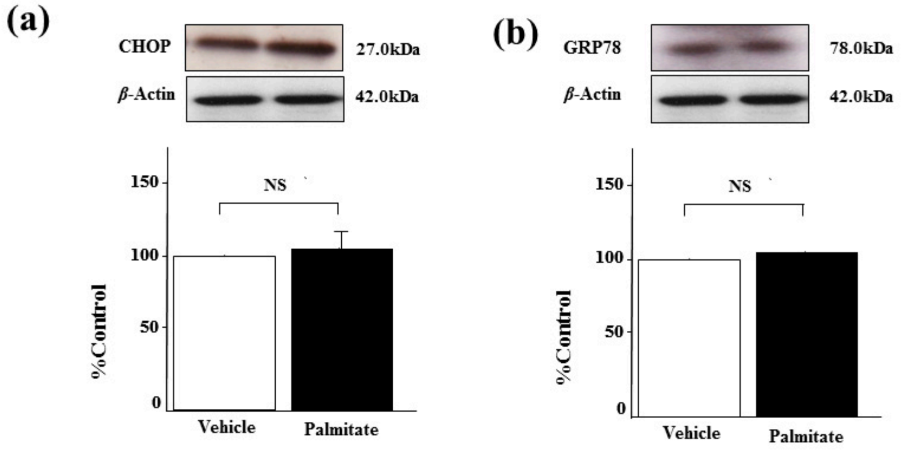

Figure S1. Palmitate (0.3 mmol/L) can not induce endoplasmic reticulum stress in hypertrophied 3T3-L1 adipocytes, in contrast to in MIN6 β-cells. Mature 3T3-L1 adipocyte were preloaded with 0.3 mmol/L palmitate or ethanol vehicle alone for 24 h (A, B). A and B: Intraceller CHOP (**a**) and GRP78 (**b**) protein content were quantified by immunoblot analysis. β-Actin served as an internal control. Results are means ± SE (n = 4). ∗∗ $p < 0.01$ compared to vehicle. NS; no significant difference compared to vehicle.
